# Evaluating effort-reward imbalance among nurses in emergency departments: a cross-sectional study in China

**DOI:** 10.1186/s12888-021-03344-6

**Published:** 2021-07-14

**Authors:** Mengge Tian, Heping Yang, Xiaoxv Yin, Yafei Wu, Guopeng Zhang, Chuanzhu Lv, Ketao Mu, Yanhong Gong

**Affiliations:** 1 Department of Social Medicine and Health Management, School of Public Health, Tongji Medical College, Huazhong University of Science and Technology, Wuhan, P. R. China; 2 Wuchang University of Technology, Wuhan, P. R. China; 3 Department of Nuclear medicine, Tongji Hospital, Tongji Medical College, Huazhong University of Science and Technology, Wuhan, P. R. China; 4 Department of Emergency, The Frist Affiliated Hospital of Hainan Medical University, Haikou, China; 5 Emergency and Trauma College, Hainan Medical University, Haikou, Hainan China; 6 Key Laboratory of Emergency and Trauma of Ministry of Education, Hainan Medical University, Haikou, Hainan China; 7 Department of Radiology, Tongji Hospital, Tongji Medical College, Huazhong University of Science and Technology, Wuhan, P. R. China

**Keywords:** Effort-reward imbalance, Emergency department, Nurses, Occupational health

## Abstract

**Background:**

Effort-reward imbalance is an adverse psychological response to working conditions that has several negative effects on nurses. However, there is little research on effort-reward imbalance and its influencing factors among nurses in emergency departments. This study aimed to understand the current situation of effort-reward imbalance and explore its influencing factors among emergency department nurses in China.

**Methods:**

From July to August 2018, a structured online questionnaire survey was conducted among emergency department nurses in China. Data were collected from emergency department nurses employed in hospitals providing pre-hospital care in China. The questionnaire consisted of sociodemographic characteristics, work-related factors and effort-reward imbalance. A descriptive analysis and a binary logistic regression were conducted to explore the effort-reward imbalance and its influencing factors among emergency department nurses.

**Results:**

The study involved 17,582 emergency department nurses; notably, the prevalence of effort-reward imbalance was 59.66%. The participating nurses who were males, aged 25 to 34 years, whose educational level was a bachelor degree or above, who had a junior or above title, who had longer years of service, and who had suffered verbal or physical violence in the past year had a higher risk of effort-reward imbalance. Furthermore, the nurses with a high monthly income, who believed that the number of nurses met the department’s demand had a lower risk of effort-reward imbalance.

**Conclusions:**

Effort-reward imbalance was prevalent among emergency department nurses in China. Measures such as adjusting the night shift frequency, increasing the number of nurses, raising salaries and reducing workplace violence should be considered to reduce the level of effort-reward imbalance.

**Supplementary Information:**

The online version contains supplementary material available at 10.1186/s12888-021-03344-6.

## Background

Nurses in emergency departments (EDs) are vital human resources within the emergency medical service system. However, because most patients admitted to EDs are urgent, critical and severe, and work in an ED is complex, varied and unpredictable, ED nurses often experience high levels of job stress [1]. Correspondingly, studies have shown that the job stress of ED nurses is higher than that of nurses in general wards [2, 3].

The effort-reward imbalance (ERI) model is a mainstream model to explain job stress, and has been widely applied to nurses in many countries [4, 5]. Accordingly, two studies, including 257 nurses in Egypt and 389 nurses in Germany, showed that 72.5 and 20.7% experienced ERI, respectively [6, 7]. In a study of 1077 nurses in China’s Shandong Province, the rate was 26.5% [8]. This diversity might be related to differences in socioeconomic development and medical systems in countries and regions. So far, most research on ERI has focused on general ward nurses, while studies involving ED nurses are less prevalent. However, ED nurses may experience more severe ERI because of the complex work environment in EDs [1]. In a study conducted on ED nurses from the United States, approximately 93% of them had an ERI [9]. High-effort/low-reward conditions at work are a hostile environment for employees, generating a series of adverse effects on employees and their work attitude [10]. Some studies have revealed that ERI is associated with depression, poor health, and cardiovascular diseases [11–13]. In addition, it could lead to a decrease in employees’ job satisfaction and an increase in turnover intention [4, 14–17]. Thus, it is necessary to explore the situation of ERI and its related factors among ED nurses.

In China, emergency medicine developed later and the emergency medical service system was not yet mature. In the background of ED overcrowding and sharply increasing demands for emergency services [18], Chinese ED nurses may have been facing a more serious situation of ERI. However, no study has been undertaken in China concerning ERI among ED nurses. Therefore, this study aimed to describe ED nurses’ current situation of ERI, and explore its influencing factors in China through a large cross-sectional survey.

## Methods

### Study design and data collection

Our study was part of a large nationwide study on the health and working conditions of ED staff in China, which was conducted from July to August 2018. This cross-sectional study was an online survey undertaken in coordination with the Medical Administration Bureau of the National Health Commission of the People’s Republic of China. In hospitals providing pre-hospital care in China, this questionnaire was distributed on the ED nurses’ work platform and administrated by an online survey platform (platform name: Questionnaire Star, website: https://www.wjx.cn). Finally, the questionnaire link was clicked by 25,518 ED nurses, who were thought to be invited into our study. Of them, 17,582 nurses completed the online questionnaire, with a response rate of 68.9%.

### Ethics statement

This study was approved by the Research Ethics Committee of Hainan Medical University, Haikou, China (protocol number: HYLL-2018-035). All the methods in this study were in accordance with the institutional research committee and tenets of the Declaration of Helsinki. All participants provided informed consent. Moreover, each individual participated in this anonymous survey voluntarily, and their information was kept confidential.

### Measurements

Data were collected using a structured anonymous questionnaire developed based on a review of the published literature [5–10]. A total of 30 ED nurses participated in a preliminary test in Wuhan. Based on the pre-test, the questionnaire was modified to ensure that the questions were clear and understandable. In this study, the questionnaire content included three parts: sociodemographic characteristics, work-related factors, and ERI. Sociodemographic characteristics and work-related factors were selected based on published literature in this field [5–9]. The ERI questionnaire used in this study was widely applied internationally, and its Chinese version has been proven to have good reliability and validity [10, 19].

The sociodemographic characteristics included sex, age, marital status and educational level. The work-related factors included professional title, hospital level, monthly income, years of service, frequency of night shifts, perceived shortage of nurses, frequency of workplace verbal violence and experiencing workplace physical violence in the past year. The perceived shortage of nurses was assessed by one question: ‘Do you think that the current number of nurses in the ED can meet daily work needs?’ (‘Does not meet demands’, ‘General’, ‘Meets demands’).

The balance between job efforts and rewards was evaluated by an effort-reward ratio (ERR), which was measured using subscales of the ERI questionnaire [10, 19]. Accordingly, six items for job efforts, including time pressure, work interruption, responsibility, extra work, physical labour, and workload increase, were rated on a five-point Likert scale ranging from 1 (strongly disagree) to 5 (strongly agree). Thus, the total score of the measured efforts ranged between 6 and 30. The higher the score, the greater the perceived effort. Eleven items for job rewards, including esteem, job promotion and job security, were rated on a five-point Likert scale ranging from 1 (strongly agree) to 5 (strongly disagree). Thus, the total score of the measured rewards ranged from 11 to 55. The higher the score, the more the perceived rewards were experienced. The ERR was calculated according to the following formula: ERR = (11 × effort)/(6 × reward). Therefore, an ERR value greater than 1.0 indicated a high level of ERI, which reflected that the invested efforts were not matched by the rewards received. In this study, the Cronbach’s alpha values of efforts and rewards were 0.82 and 0.92, respectively.

### Statistical analysis

Pearson’s chi-squared test was used to conduct descriptive analyses of sociodemographic characteristics and work-related factors in different effort-reward status groups. Categorical variables were reported as frequencies and percentages. Moreover, the associations of the independent variables with ERI were explored using binary logistic regression and a stepwise selection method. The odds ratios (ORs) and 95% confidence intervals (CIs) of the variables were reported. Spearman’s rho correlations and the variance inflation factor (VIF) were used to test for multicollinearity between independent variables. The Spearman’s rho correlations were at the maximum threshold of 0.7 and the VIF was at maximum threshold of 10 (mean VIF = 1.33, max VIF = 2.07 and minimum = 1.06), which indicated that no collinearity was detected (Supplementary Table 1). A Hosmer-Lemeshow goodness-of-fit test was used to examine model fitness (*p* = 0.309). All the analyses were conducted at a statistical significance level of 0.05, using the Statistical Analysis System (SAS) version 9.4 for Windows (SAS Institute Inc., Cary, NC, USA).

## Results

### Participants’ characteristics for sociodemographic variables and work-related factors

The basic characteristics of the ED nurses are summarised in Table 1. A total of 17,582 ED nurses completed the survey; 10.25% of them were male and 89.75% were female. The ages of the ED nurses tended to be young and 79.81% were younger than 35. In this sample, the educational level was mainly an associate degree, which accounted for 48.93% of the participants. Correspondingly, 61.81% of them were married. Moreover, in this survey, 37.33%, 39.55%, and 4.14% of the ED nurses worked in level III, level II, level I hospitals respectively, and 18.99% worked in emergency centres. In addition, Table 1 describes the different characteristics of ED nurses between the effort-reward balance and ERI groups. In this study, 59.66% of the ED nurses experienced ERI. Consequently, significant differences were found between the groups for the following items: sex, age, marital status, educational level, professional title, hospital level, years of service, perceived shortage of nurses, frequency of night shifts, workplace verbal violence and workplace physical violence (*p* < 0.05).

**Table 1:** Comparisons of characteristics in different effort-reward status groups among nurses in emergency department

	Total	Effort-reward balance	Effort-reward imbalance	χ^**2**^	***P***
	N (%)	N (%)	N (%)		
**Total**	17,582 (100)	7093 (40.34)	10,489 (59.66)		
**Sociodemographic characteristics**					
**Age**				145.78	< 0.001^**^
<25	3493 (19.87)	1722 (24.28)	1771 (16.88)		
25 ~ 34	10,540 (59.95)	4036 (56.90)	6504 (62.01)		
>34	3549 (20.19)	1335 (18.82)	2214 (21.11)		
**Sex**				32.43	< 0.001^**^
Male	1803 (10.25)	615 (8.67)	1188 (11.33)		
Female	15,779 (89.75)	6478 (91.33)	9301 (88.67)		
**Educational level**				100.29	< 0.001^**^
Vocational diploma or less^a^	1105 (6.28)	534 (7.53)	571 (5.44)		
Associate degree^b^	8603 (48.93)	3686 (51.97)	4917 (46.88)		
Bachelor degree or above	7874 (44.78)	2873 (40.50)	5001 (47.68)		
**Marital status**				55.96	< 0.001^**^
Unmarried/other	6714 (38.19)	2945 (41.52)	3769 (35.93)		
Married	10,868 (61.81)	4148 (58.48)	6720 (64.07)		
**Work-related factors**					
**Hospital level**				11.33	0.010^*^
Emergency centres	3338 (18.99)	1343 (18.93)	1995 (19.02)		
Level I hospitals	728 (4.14)	333 (4.69)	395 (3.77)		
Level II hospitals	6953 (39.55)	2828 (39.87)	4125 (39.33)		
Level III hospitals	6563 (37.33)	2589 (36.50)	3974 (37.89)		
**Title**				105.66	< 0.001^**^
None	2328 (13.24)	1116 (15.73)	1212 (11.55)		
Junior	11,713 (66.62)	4754 (67.02)	6959 (66.35)		
Intermediate or above	3541 (20.14)	1223 (17.24)	2318 (22.10)		
**Monthly income (YUAN)**				3.3	0.348
≤ 2499	2980 (16.95)	1236 (17.43)	1744 (16.63)		
2500 ~ 4000	7583 (43.13)	3075 (43.35)	4508 (42.98)		
4001 ~ 6000	4649 (26.44)	1849 (26.07)	2800 (26.69)		
≥ 6001	2370 (13.48)	933 (13.15)	1437 (13.70)		
**Years of service**				268.48	< 0.001^**^
< 1	2540 (14.45)	1360 (19.17)	1180 (11.25)		
1–5	7783 (44.27)	3183 (44.88)	4600 (43.86)		
6–10	4573 (26.01)	1581 (22.29)	2992 (28.53)		
≥ 11	2686 (15.28)	969 (13.66)	1717 (16.37)		
**Frequency of night shifts (per month)**				242.99	< 0.001^**^
0 ~ 5	5417 (30.81)	2537 (35.77)	2880 (27.46)		
6 ~ 10	7719 (43.90)	3122 (44.02)	4597 (43.83)		
11 ~ 15	3651 (20.77)	1243 (17.52)	2408 (22.96)		
≥ 16	795 (4.52)	191 (2.69)	604 (5.76)		
**Shortage of nurses**				933.82	< 0.001^**^
Does not meet demands	8834 (50.24)	2435 (34.33)	6399 (61.01)		
General	5231 (29.75)	2565 (36.16)	2666 (25.42)		
Meets demands	3517 (20.00)	2093 (29.51)	1424 (13.58)		
**Workplace verbal violence (times)**				1300.51	< 0.001^**^
0	5351 (30.43)	2975 (41.94)	2376 (22.65)		
1 ~ 3	7369 (41.91)	2932 (41.35)	4437 (42.30)		
≥ 4	4862 (27.65)	1186 (16.72)	3676 (35.05)		
**Workplace physical violence**				265.11	< 0.001^**^
Yes	2357 (13.41)	590 (8.32)	1767 (16.85)		
No	15,225 (86.59)	6503 (91.68)	8722 (83.15)		

^*^ *P* value < 0.05, ^**^ *p* value < 0.001

^a^A vocational diploma refers that students graduating from senior middle school receive 2 years of education in vocational schools, or students graduating from junior middle school receive 3 years of education in vocational schools

^b^An associate degree refers that students graduating from senior middle school (grade year 10 to year 12) receive 3 years of education in college, or students graduating from junior middle school (grade year 7 to year 9) receive 5 years of education in college

### Effect of sociodemographic variables and work-related factors on ERI

Table 2 presents the relationships between ERI and sociodemographic and work-related factors. The risk of experiencing ERI was higher among ED nurses aged 25 to 34 than among those aged less than 25 (OR = 1.13, 95% CI: 1.03–1.24). Our results also revealed that when ED nurses were male (OR = 1.24, 95% CI: 1.11–1.39), had a bachelor’s degree or above (OR = 1.21, 95% CI: 1.05–1.39), junior professional title (OR = 1.13, 95% CI:1.02–1.25), and intermediate or above professional title (OR = 1.43, 95% CI: 1.24–1.65), they had higher odds of experiencing ERI. ED nurses with a higher monthly income were less likely to exhibit ERI (OR = 0.73, 95% CI: 0.64–0.82). Reverse trends were observed for years of service and the frequency of night shifts. ED nurses who had longer years of service (OR = 1.48, 95% CI: 1.28–1.71) and had a higher frequency of night shift (OR = 1.93, 95% CI: 1.61–2.31) were more likely to experience ERI. Compared to perceiving a shortage of nurses, when the number of nurses met the demands of the department, the risk of ERI among them markedly decreased (OR = 0.32, 95% CI: 0.30–0.35). In addition, compared to not having suffered verbal violence in the last year, the rate of the occurrence of ERI increased 0.64 times and 1.72 times when ED nurses had suffered workplace verbal violence one to three times (OR = 1.64, 95% CI: 1.52–1.76) and four times or more (OR = 2.72, 95% CI: 2.48–2.99), respectively. Analogously, ED nurses who had suffered workplace physical violence in the last year were 1.32 times more likely to experience ERI than those who had not (OR = 1.32, 95% CI: 1.19–1.48).

**Table 2:** Binary logistic regression examining factors associated with ERI

	***OR (95%CI)***	***P***
**Age**		
< 25	1.00(reference)	
25 ~ 34	1.13 (1.03–1.24)	0.016^*^
> 34	1.03 (0.90–1.19)	0.653
**Sex**		
Male	1.24 (1.11–1.39)	< 0.001^**^
Female	1.00(reference)	
**Education level**		
Vocational diploma or less^a^	1.00(reference)	
Associate degree^b^	1.12 (0.97–1.28)	0.113
Bachelor degree or above	1.21 (1.05–1.39)	0.009^*^
**Title**		
None	1.00(reference)	
Junior	1.13 (1.02–1.25)	0.015^*^
Intermediate or above	1.43 (1.24–1.65)	< 0.001^**^
**Monthly income (YUAN)**		
≤ 2499	1.00(reference)	
2500 ~ 4000	0.84 (0.76–0.92)	< 0.001^**^
4001 ~ 6000	0.76 (0.69–0.85)	< 0.001^**^
≥ 6001	0.73 (0.64–0.82)	< 0.001^**^
**Years of service**		
< 1	1.00(reference)	
1–5	1.34 (1.21–1.48)	< 0.001^**^
6–10	1.58 (1.40–1.78)	< 0.001^**^
≥ 11	1.48 (1.28–1.71)	< 0.001^**^
**Frequency of night shifts (per month)**		
0 ~ 5	1.00(reference)	
6 ~ 10	1.09 (1.01–1.18)	0.028^*^
11 ~ 15	1.29 (1.17–1.41)	< 0.001^**^
≥ 16	1.93 (1.61–2.31)	< 0.001^**^
**Shortage of nurses**		
Does not meet demands	1.00(reference)	
General	0.46 (0.42–0.49)	< 0.001^**^
Meets demands	0.32 (0.30–0.35)	< 0.001^**^
**Workplace verbal violence (times)**		
0	1.00(reference)	
1 ~ 3	1.64 (1.52–1.76)	< 0.001^**^
≥ 4	2.72 (2.48–2.99)	< 0.001^**^
**Workplace physical violence**		
Yes	1.32 (1.19–1.48)	< 0.001^**^
No	1.00(reference)	

^*^P value < 0.05, ^**^p value < 0.001, Hosmer and Lemeshow goodness of fit test (*p*-value = 0.309)

^a^A vocational diploma refers that students graduating from senior middle school receive 2 years of education in vocational schools, or students graduating from junior middle school receive 3 years of education in vocational schools

^b^An associate degree refers that students graduating from senior middle school (grade year 10 to year 12) receive 3 years of education in college, or students graduating from junior middle school (grade year 7 to year 9) receive 5 years of education in college

## Discussion

This study found that about 60% of ED nurses experienced ERI, which reflected that the current situation of psychological effects caused by poor work conditions was not optimistic. This result was higher than the ratio of 26.5% in a study that investigated the prevalence of ERI among 1077 nurses in Shandong Province [8], which revealed that the problem of experiencing ERI was more serious for ED than general ward nurses. The probable reason might be that compared to general wards, the ED was mainly for patients with acute and critical illnesses, and therefore, the nurses undertook more intensive and risky tasks and were in a more severe work environment [20]. A previous study showed that 40.1% of nurses witnessed the occurrence of medical errors at least once [21]. The fear of medical errors could exacerbate nurses’ doubts about self-competence, which might make them pay more attention and bear more psychological pressure in their nursing work [22, 23].

Our study found that ED nurses aged 25 to 34 had a higher incidence of ERI compared to those aged less than 25. However, there was no significant increase in the risk of ERI among ED nurses aged above 34. According to a previous study on continuing professional development of nurses, younger nurses started out in their careers and were still in the stage of professional development by acquiring work experience [24]. They were full of fervour and longing to work and family pressure was relatively less. Middle-aged nurses enjoyed a period of prosperity in their career development. In addition to trying to improve their self-ability, they needed to deal with various matters from their families as well as from society. Therefore, balancing life at home with work has become a huge challenge at this stage [24]. These could be the factors that led ED nurses aged 25 to 34 to have a higher risk of ERI. Another study on clinical competence among Korean nurses showed that with their wealth of work experience, older nurses had improved their professional knowledge, skills and work efficiency, and their work and life gradually became increasingly stable [24, 25]. Thus, there was no significant increase in perceived risk of ERI.

There was a correlation between gender and the occurrence of ERI [26]. Our study showed that male ED nurses were more likely to experience ERI than the female nurses, which might be because male nurses often undertook more work in clinical practice [27]. A previous study in China also indicated that male nurses took heavy workloads and experienced great mental stress [28]. Nevertheless, the contemporary nursing profession is still dominated by women, and men tend to be rejected in nursing work because of the gender stereotype, which might lead to male nurses investing high efforts and receiving low rewards [29]. In addition, our study indicated that ED nurses who had a high educational level (a bachelor’s degree or above) were at a higher risk of ERI. Studies have shown that although highly educated nurses have made more efforts in learning, they still undertake the same work with low-educated nurses in clinical nursing in China, which may be the reason why they are prone to ERI [30, 31].

There was a close relationship between many work-related factors and ERI among ED nurses. The results of our study showed that ED nurses who perceived a shortage of nurses, had a high night shift frequency and a low salary were at a higher risk of ERI. A perceived shortage of nurses and a high night shift frequency indicated that ED nurses were in a state of high efforts, while a low salary indicated inadequate rewards. This is consistent with previous studies [32–35]. ED nurses who had longer years of service were at a higher risk of ERI, which might be related to their long-term commitment to high-intensity work responsibilities [36]. In China, ED nurses with professional titles are more likely to expose themselves to ERI because they tend to undertake more work tasks and responsibilities [34]. Moreover, workplace violence was also an important factor associated with ERI among nurses in EDs. This was related to the psychological resistance of nurses to work caused by workplace violence, which increased their psychological and work stress [37]. Therefore, administrators should realise the importance of working conditions and take relevant measures, such as a reasonable arrangement of shifts, timely adjustments of staff allocation and salary levels, and building a safe working environment to reduce the incidence of ERI for ED nurses.

### Strengths and limitations

This was a national cross-sectional study with a large sample size. To the best of our knowledge, this study is the first to investigate the ERI and its influencing factors among ED nurses in China. In addition, there were some limitations to the findings. First, our study focused only on ED nurses, and our findings should be carefully generalised to other populations. Second, it was based on convenience sampling, which may have a selection bias. It is also expected that nurses who were more interested in the study subject were more likely to participate in the survey, which may have resulted in an overestimation of ERI levels. However, as this study was a nationwide and large sample survey, the sample should be representative to some extent. Third, it is not possible to draw a causal relationship because of the cross-sectional nature of the study. Therefore, further prospective studies are required.

## Conclusions

Overall, there was a high prevalence of ERI among ED nurses in China. Based on the study’s results, administrators should emphasise male ED nurses aged 25 to 34, with an educational background of a bachelor’s degree or above and a junior, intermediate or above title, and take targeted measures to reduce their level of ERI. On the one hand, it is necessary to adjust the frequency of night shifts and increase the number of nurses to reduce ED nurses’ sense of effort. On the other hand, the income of ED nurses should be appropriately increased, and a safe work environment should be created to reduce the occurrence of workplace violence to increase their sense of rewards attained from work.

## Supplementary Information

**Additional file 1: Table S1.** Correlation between included variables.

## Data Availability

The datasets used and/or analysed during the current study are available from the corresponding author on reasonable request.

## Abbreviations

ED: Emergency department
ERI: Effort-reward imbalance
ERR: Effort-reward ratio
OR: Odds ratios
95% CI: 95% confidence intervals
VIF: Variance inflation factor
