# Supplementary material for: Evaluating effort-reward imbalance among nurses in emergency departments: a cross-sectional study in China
Source: BMC Psychiatry. 2021 Jul 14;21:353. doi: 10.1186/s12888-021-03344-6 (PMC8278678; doi:10.1186/s12888-021-03344-6)
Supplement: Supplementary file 1 — Additional file 1: Table S1. Correlation between included variables. [file 12888_2021_3344_MOESM1_ESM.docx]

**Supplementary Table 1. correlation between included variables**

|  | 1 | 2 | 3 | 4 | 5 | 6 | 7 | 8 | 9 | 10 |
| --- | --- | --- | --- | --- | --- | --- | --- | --- | --- | --- |
| 1. Age | 1 | 0.119^**^ | 0.233^**^ | 0.541^**^ | 0.268^**^ | 0.583^**^ | -0.078^**^ | -0.112^**^ | 0.031^**^ | -0.011 |
| 2. Sex |  | 1 | -0.005 | 0.098^**^ | -0.043^**^ | 0.076^**^ | -0.114^**^ | -0.006 | -0.076^**^ | 0.155^**^ |
| 3. Education level |  |  | 1 | 0.243^**^ | 0.252^**^ | 0.216^**^ | 0.003 | -0.068^**^ | 0.097^**^ | -0.063^**^ |
| 4. Title |  |  |  | 1 | 0.282^**^ | 0.430^**^ | -0.080^**^ | -0.089^**^ | 0.039^**^ | -0.017^*^ |
| 5. Monthly income |  |  |  |  | 1 | 0.282^**^ | -0.024^*^ | -0.028^**^ | 0.064^**^ | 0.004 |
| 6. Years of service |  |  |  |  |  | 1 | -0.010 | -0.103^**^ | 0.106^**^ | -0.039^**^ |
| 7. Frequency of  night shifts |  |  |  |  |  |  | 1 | -0.119^**^ | 0.163^**^ | -0.114^**^ |
| 8. Shortage of nurses |  |  |  |  |  |  |  | 1 | -0.198^**^ | 0.092^**^ |
| 9. Workplace verbal  violence |  |  |  |  |  |  |  |  | 1 | -0.311^**^ |
| 10. Workplace physical  violence |  |  |  |  |  |  |  |  |  | 1 |

^**^. Correlation is significant at the 0.01 level (2-tailed). ^*^. Correlation is significant at the 0.05 level (2-tailed).
